# Comparative Sequence Analyses of La Crosse Virus Strain Isolated from Patient with Fatal Encephalitis, Tennessee, USA 

**DOI:** 10.3201/eid2105.141992

**Published:** 2015-05

**Authors:** Amy J. Lambert, Rebecca Trout Fryxell, Kimberly Freyman, Armando Ulloa, Jason O. Velez, Dave Paulsen, Robert S. Lanciotti, Abelardo Moncayo

**Affiliations:** Centers for Disease Control and Prevention, Fort Collins, Colorado, USA (A.J. Lambert, J.O. Velez, R.S. Lanciotti);; University of Tennessee, Knoxville, Tennessee, USA (R.T. Fryxell, D. Paulsen);; Tennessee Department of Health, Nashville, Tennessee, USA (K. Freyman, A. Ulloa, A. Moncayo)

**Keywords:** La Crosse virus, viruses, sequence, Tennessee, children, vector-borne infections, mosquitoes, encephalitis, United States

## Abstract

We characterized a La Crosse virus (LACV) isolate from the brain of a child who died of encephalitis-associated complications in eastern Tennessee, USA, during summer 2012. We compared the isolate with LACV sequences from mosquitoes collected near the child’s home just after his postmortem diagnosis. In addition, we conducted phylogenetic analyses of these and other sequences derived from LACV strains representing varied temporal, geographic, and ecologic origins. Consistent with historical findings, results of these analyses indicate that a limited range of LACV lineage I genotypes is associated with severe clinical outcomes.

## Abstract 2

We verified the transovarial maintenance and ecologic role of the endemic vector in this region.

La Crosse virus (LACV) (family *Bunyaviridae*, genus *Orthobunyavirus*) is the primary cause of arthropod-borne viral (arboviral) encephalitis in children in the United States. LACV has a genome of 3 negative-stranded RNA segments (small, medium, and large) and is endemic to forested regions along the Mississippi and Ohio River basins, east of the Rocky Mountains (*1*–*5*). These forests provide a habitat for the known principal vector of LACV, *Aedes triseriatus* mosquitoes*.* Amplifying hosts include small mammals that develop levels of viremia sufficient to transmit LACV to mosquitoes during the summer months. Human infections occur during the summer and early fall, when humans are at greatest risk for mosquito bites.

In recent years, LACV reportedly has increased above endemic levels in regions of the southeastern United States, including areas of eastern Tennessee (*6*). The reason for this apparent increase remains unknown; possible causes include a change in transmission dynamics contributed to by invasive vector species or emergence of a relatively virulent strain of LACV in those regions. Confounding a better understanding of the latter, LACV has been historically difficult to isolate from humans. In fact, just 3 human isolates, derived over >50 years, were described in GenBank (accession nos. EF485033–35, EF485030–32, GU206139) before our study began.

We report the multisegment genomic characterization of an LACV strain isolated from the brain of a child who died of encephalitis-associated complications in eastern Tennessee, USA, in July 2012. To the best of our knowledge, this represents only the fourth human isolate of LACV that has been described at the nucleotide sequence level. In addition, we have determined the coding sequences of LACV strains derived from mosquitoes reared from eggs collected within 16 radial kilometers (10 miles) of the child’s home during summer 2012, after the postmortem diagnosis.

## Case Description

In July 2012, a 6-year-old boy from Union County, Tennessee, was seen at an emergency department after 2 focal seizures and symptoms consistent with viral encephalitis. Because of the geographic location, seasonal timing of illness, and clinical presentation, LACV was suspected as the cause of illness. A serum sample was determined to be immunofluorescence assay–positive for LACV IgM and IgG. On day 2 after admission, the boy displayed altered mental status, decreased mobility, hallucinations, and vomiting. By day 3, he was responsive only to painful stimuli and showed decreased arousal. His condition progressively deteriorated, and he was pronounced brain dead and died on day 5 after admission. At autopsy, a section of the temporal lobe was taken, immediately frozen, and sent to the Centers for Disease Control and Prevention (CDC), Division of Vector-Borne Diseases (Fort Collins, CO, USA), for analysis.

## Materials and Methods

At CDC, the sample was determined to be LACV-positive by real-time reverse transcription PCR (RT-PCR) by using previously described methods (*7*). This result was confirmed by next-generation sequencing by using the Ion Torrent PGM system (Life Technologies, Grand Island, NY, USA) and methods described elsewhere (*8*). After nucleic acid sequence detection, the brain sample was subjected to a standard isolation method in an attempt to derive an LACV isolate. Briefly, 200 μL of 10^0^ and 10^−1^ dilutions of supernatant taken from a homogenized preparation of the temporal lobe sample were inoculated onto confluent Vero cells in T25 flasks. The flasks were then incubated at 37°C and reviewed daily for cytopathic effect. Cytopathic effect was identified in flasks inoculated with both the 10^0^ and 10^−1^ dilutions on day 4 postinoculation. LACV was confirmed in the supernatants collected from these flasks by real-time RT-PCR.

To detect LACV in the geographic area, oviposition, BG sentinel traps, (Bioagents AG, Regensburg , Germany; and CDC light and gravid traps, (Clarke, St. Charles, IL, USA), were placed at 49 sites consisting of cemeteries and houses within 10 miles of the patient’s home (the presumed site of infection). Traps were set during September 5–October 3, 2012. A total of 816 egg papers were collected, and on arrival at the Tennessee Department of Health, eggs were counted and each paper flooded 3 times with deionized water. Egg papers were dried, and eggs were allowed to embryonate for 1 week in a humidified container. Each egg paper was placed in a plastic tray (28 × 11 × 12 cm) containing 0.5–1 L of deionized water, and emerging larvae were fed bovine liver powder ad libitum (#02900396 MP Biomedicals, Solon, OH, USA). Each tray was placed into an incubator at 26 ± 2°C, 60%–80% relative humidity, and a 12-h light–12-h dark photoperiod. Pupae were collected by using plastic pipettes and transferred into small bowls containing 0.1 L of deionized water that were housed in 60-mL screened cages for adult emergence. Adults were removed by using hand–held insect vacuums and placed in another chamber, frozen, and then morphologically identified to species and sex (*9*). Adults identified from the field or reared from egg papers were pooled in cohorts of <25 specimens of the same species, sex, trap, date, and site.

Each pool of mosquitoes was combined with 3 copper BBs and 1 mL of Eagle’s minimum essential medium with 2% fetal bovine serum, 0.5% NaHCo_3_, and 1% antimicrobial solution (10,000 IU/mL penicillin, 10,000 μg/mL streptomycin, 25 μg/mL amphotericin B; Sigma-Aldrich Co., St. Louis, MO, USA). The samples were homogenized on a Retsch MM300 shaker (Retsch Gmbh & Co. KG, Haan, Germany) for 90 sec, centrifuged at 5,000 rpm for 5 min, and stored at –80°C. RNA was extracted from 200 μL of each sample by using the QIAamp Viral Isolation 96-well protocol on the BioRobot 9604 or the QIAamp Viral RNA Mini Kit (QIAGEN, Valencia, CA, USA), following the manufacturer’s protocol.

All samples were tested for orthobunyavirus small segment RNA by RT-PCR by using published primers from Kuno et al (*10*) (forward BCS82C 5′-ATGACTGAGTTGGAGTTTCATGATGTCGC-3′, reverse BCS332V 5′-TGTTCCTGTTGCCAGGAAAAT-3′). Each 25-μL reaction contained 11.25 μL of nuclease-free water, 2.5 μL of 10× RT-PCR buffer, 200 μM dNTPs, 2 U/μL of RT, 5 U/μL of Easy-A High Fidelity DNA polymerase (Agilent Technologies, Santa Clara, CA, USA), 50 pmol of each primer, and 5 μL of extracted RNA. We conducted RT-PCR in the GeneAmp PCR system 9700 (Applied Biosystems) with the following cycle times and temperatures: 1 cycle at 45°C for 30 min; 1 cycle at 95°C for 10 min; 45 cycles of 95°C for 30 s, 55°C for 60 s, 68°C for 120 s; and 1 cycle at 68°C for 5 min. RT-PCR products were visualized by using the E-Gel electrophoresis system (Life Technologies). The positive control originated from a pool of 10 *Ae. triseriatus* mosquitoes collected from Fulton County, Georgia, USA, in 2002. RNAase-free deionized water was used as a negative RT-PCR control.

Products of positive RT-PCR reactions were gel purified by using the QIAquick Gel Extraction Kit protocol (QIAGEN) and then sequenced at the Tennessee Department of Health Laboratory Services (Nashville, TN, USA) with a 3130 × 1 genetic sequencer (Applied Biosystems) by using the BigDye Terminator v1.1 Cycle Sequencing Kit (Applied Biosystems). We then analyzed the resulting sequences using Sequencer 4.6 software (Gene Codes Corp., Ann Arbor, MI, USA) and compared them with sequences in GenBank by BLAST analyses (http://blast.ncbi.nlm.nih.gov/Blast.cgi) for identification. Samples with a >96% identity with other published LACV sequences were considered preliminarily identified as LACV positive.

To confirm results from multiple genomic segments, we reextracted RNA from all LACV-positive pools and retested them using real-time RT-PCR with a second published primer set targeting the medium segment of the viral genome (*7*). Using these methods, we determined that 4 pools of *Ae. triseriatus* mosquitoes were LACV positive. The positive pools came from egg papers collected at a cemetery on October 3, 2012.

We generated multisegment genomic sequence data from the newly derived LACV human isolate, along with the 4 LACV-positive *Ae. triseriatus* mosquitoes (2 male and 2 female), by next-generation sequencing methods conducted as previously described (*8*). Results were confirmed by spot sequencing using traditional Sanger methods, as described elsewhere (*8*).

After nucleotide sequence level characterization of the Tennessee 2012 LACV strains of both human and mosquito origin, we conducted comparative sequence and phylogenetic analyses using MEGA5 software and methods described previously (*11*). Phylogenetic methods were applied to 2 representative Tennessee 2012 *Ae. triseriatus* mosquito strains, along with the Tennessee 2012 human sequence and a diverse set of data from GenBank (Figure). GenBank accession numbers for sequences described in our study are KP271104–KP271118.

**Figure:**
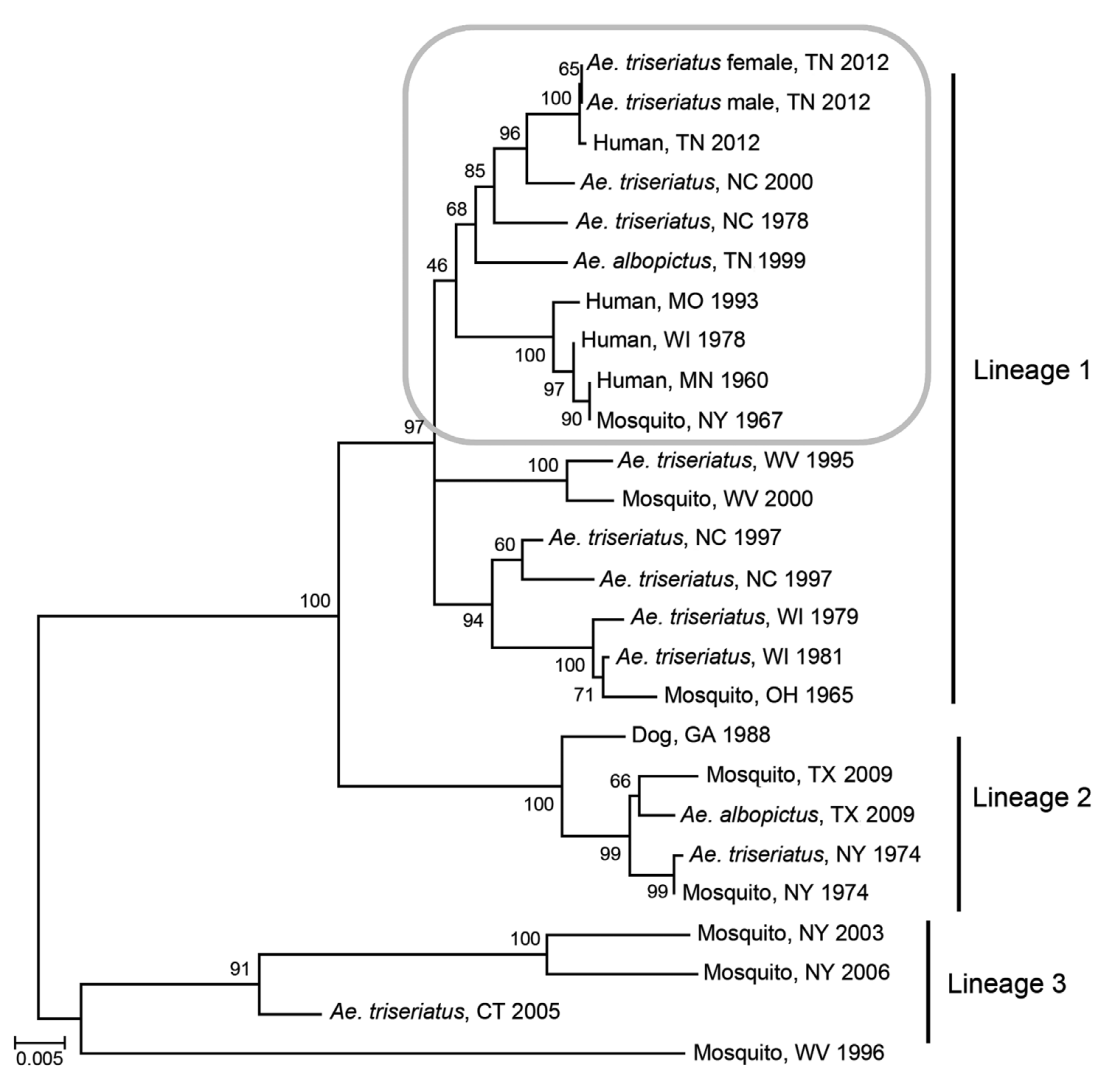
Phylogeny of medium segment sequences of selected La Crosse virus strains of varied temporal, geographic, and ecological origin. Taxon descriptions are restricted in some cases according to a limited amount of information in GenBank. A neighbor-joining method was used with 2,000 replicates for bootstrap testing. Scale bar represents 0.005 nt substitutions per site. Box indicates the area of phylogenetic interest.

## Results and Discussion

The 4 LACV strains derived from *Ae. triseriatus* mosquito eggs were highly similar to the patient’s isolate, with >99%, >97%, and >98% nt sequence identities shared among compared small, medium, and large segment sequences, respectively. The Tennessee 2012 LACV strains grouped with strong support in lineage I, along with all described human isolates of LACV from varied geographic and temporal origins (Figure). This finding is consistent with results from analyses of more limited historical datasets (*12*,*13*), including data generated from direct amplification of LACV cDNA from central nervous system tissues of persons who died (*14*) and indicates that a restricted range of LACV genotypes is associated with severe clinical outcomes. In addition, a sequence from GenBank that was generated from *Ae. triseriatus* mosquitoes collected in North Carolina during summer 2000 grouped with strong support along with the Tennessee 2012 human and mosquito strains (Figure). Coincidentally, we analyzed samples from a child from North Carolina who, during summer 2000, had fatal LACV-associated encephalitis (A.J. Lambert, unpub. data). At that time, we were unable to derive an isolate or generate sequence data because of the low levels of LACV, as inferred by real-time RT-PCR, in those samples (A.J. Lambert, unpub. data). The presence of a strain highly similar to the Tennessee isolates in a vector mosquito collected at the same general time and location of the fatal case in North Carolina during 2000 suggests that a Tennessee 2012–like lineage I genotype also might have been responsible for the North Carolina case.

Taken together, our results confirm the epidemiologic importance of LACV lineage I strains in the emergence of fatal LACV disease throughout broad temporal and geographic ranges, including the southeastern United States. Furthermore, identification of LACV lineage I strains in male *Ae. triseriatus* mosquitoes collected near the presumed site of infection in this case verifies transovarial maintenance and the ecologic importance of the endemic vector in Tennessee. Our findings justify further studies, designed to discriminate possible differences in the ecology, epidemiology, and biology of LACV strains from varied phylogenetic lineages.
